# Out-of-hospital cardiac arrest patients during the coronavirus disease 2019 pandemic

**DOI:** 10.1038/s41598-023-50150-z

**Published:** 2023-12-27

**Authors:** Kenta Watanabe, Kosuke Mori, Kosuke Sato, Takeru Abe, Shouhei Imaki, Ichiro Takeuchi

**Affiliations:** 1 Department of Emergency Medicine, Yokohama Municipal Citizen’s Hospital, Yokohama City, Kanagawa Japan; 2 Department of Emergency Medicine, Yokohama City University, Yokohama City, Kanagawa Japan; 3 Medical Center Advanced Critical Care and Emergency Center, Yokohama City University, Yokohama City, Kanagawa Japan

**Keywords:** Respiratory distress syndrome, Viral infection, Risk factors

## Abstract

The coronavirus disease 2019 (COVID-19) pandemic had severe impact on the outcome of out-of-hospital cardiac arrest (OHCA) patients and the possibility of bystander cardiopulmonary resuscitation (CPR). Previous studies focused only on the short periods of the pandemic and reported a significant increase in the number of infections. In a retrospective cohort study we aimed to compare the outcomes of OHCA patients 1 year before and 1 year after the onset of COVID-19. Data of 519 OHCA patients during the pre-pandemic (January–December 2019; 262 patients) and intra-pandemic (April 2020–March 2021; 257 patients) periods in Yokohama Municipal Hospital, Japan were collected and analysed retrospectively. The study outcomes were the return of spontaneous circulation (ROSC), admission to hospital, survival to discharge, and cerebral performance category at discharge. The intra-pandemic period was associated with decreased bystander CPR (*P* = 0.004), prolonged transport time (*P* < 0.001), delayed first adrenaline administration (*P* < 0.001), and decrease in ROSC (*P* = 0.023). Logistic regression analysis revealed that the following factors were significantly associated with ROSC: “pandemic”, “shockable initial waveform”, and “witness presence”.

## Introduction

In September 2021, the city of Yokohama, with a total population of 3,778,876, has experienced the most explosive outbreak since the beginning of the COVID-19 pandemic in Japan, with a cumulative total of 65,585 infected people and 490 deaths (up to 10 September 2021)^1^. The coronavirus disease 2019 (COVID-19) outbreak resulted in high infection and mortality rates worldwide. The COVID-19 pandemic also affected out-of-hospital cardiac arrest (OHCA) patients. Previous studies have highlighted reduced return of spontaneous circulation (ROSC) rates and survival rates in patients with OHCA during the pandemic^2–6^. Five elements are critical to the survival of patients with OHCA: a call to emergency medical services (EMS), immediate and high-quality cardiopulmonary resuscitation (CPR), early defibrillation, EMS transport and advanced resuscitation, and treatment after resumption of cardiac rhythm^7^. Observational studies in COVID-19 endemic areas have noted an increase in OHCA, a decrease in the occurrence of outdoor cardiac arrest, a decrease in bystander CPR and automated external defibrillator (AED) use, and an increase in EMS response time^3–5,8–11^. However, previous studies have focused only on the short periods of the pandemic, during which there were significant increases in the number of infections.

In this study, we aimed to compare the outcomes of patients with OHCA who were transported to Yokohama Municipal Hospital, a tertiary emergency hospital in Yokohama City, 1 year before and 1 year after the onset of the COVID-19 pandemic.

## Results

**Figure 1:**
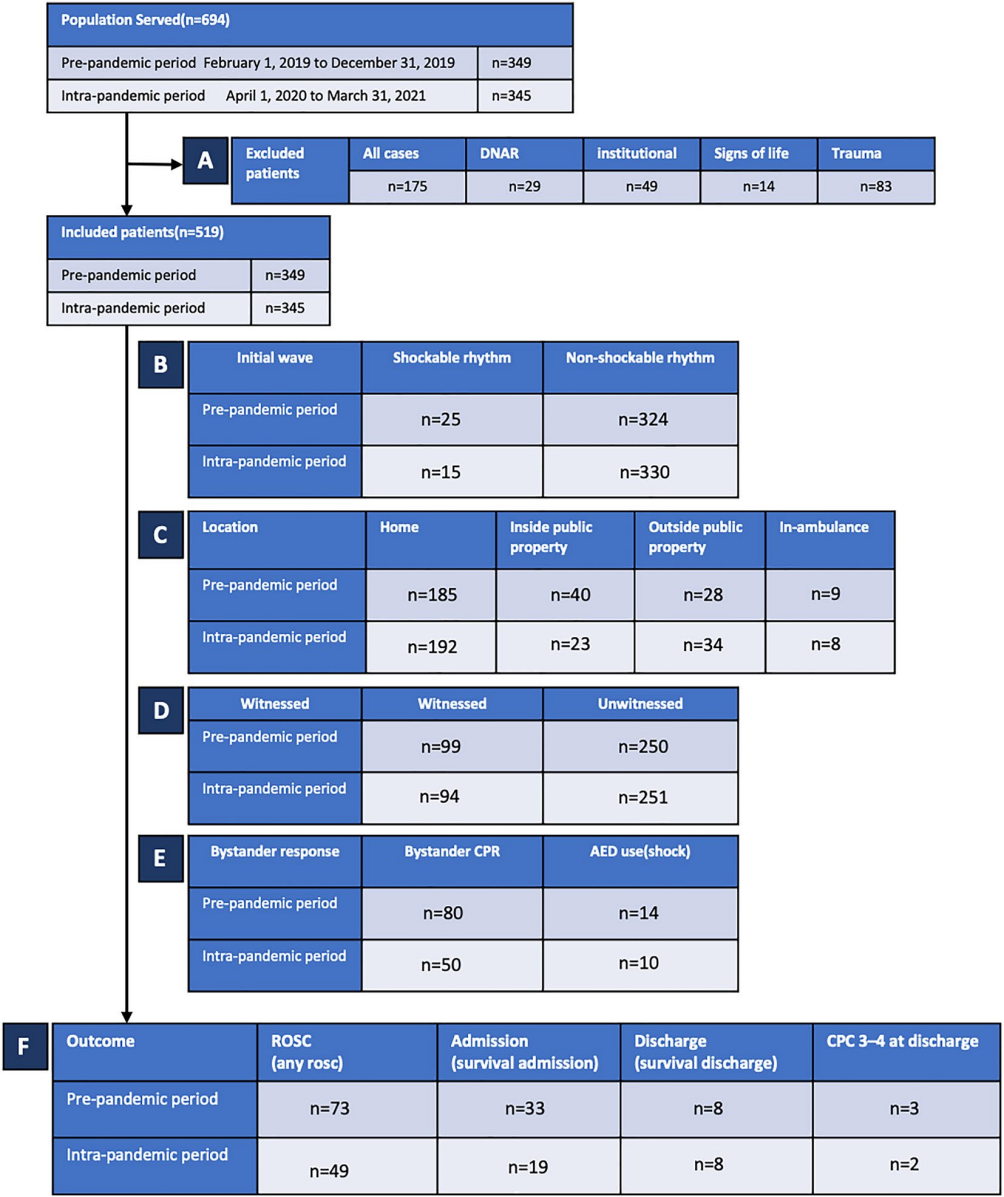
OHCA Patients in Yokohama City during (1 April 2020 to 31 March 2021) and before (1 February 2019 to 31 December 2019) the COVID-19 pandemic. *OHCA* out-of-hospital cardiac arrest, *DNAR* do not attempt resuscitation, *COVID-19* coronavirus disease 2019, *CPR* cardiopulmonary resuscitation. (**A**) Excluded patients. (**B**–**E)** Initial wave form, Location, Witnessed arrest and bystander response of included patients. (**F**) Outcomes after out-hospital cardiac arrest of included patients.

During the study period, 694 OHCA patients were transported to our hospital; of these, 175 patients (DNAR [n = 29], institutional cardiac arrest [n = 49], non-cardiac arrest [n = 14], and trauma [n = 83]) were excluded. In total, 519 patients were included in the study (262 and 257 in the pre-pandemic and intra-pandemic periods, respectively; Fig. 1).

Table 1 shows the patients’ backgrounds and outcomes; there was a statistically significant decrease in bystander CPR, prolongation of transport time (9.6% increase), and delay in first adrenaline administration (13.2% increase) during the intra-pandemic, compared to that in the pre-pandemic period. When bystander CPR rates were compared by location, only OHCA occurring at home was significantly lower during the intra-pandemic period. As an outcome, there was a statistically significant decrease in ROSC rate during the intra-pandemic period. There were no statistically significant differences in other outcomes (survival at admission, survival to discharge, and cerebral performance category (CPC) at discharge).

**Table 1:** Characteristics and outcomes of OHCA patients before and during the COVID-19 pandemic.

Factor	All patients	Pre-pandemic period (n = 262)	Intra-Pandemic period (n = 257)	*P* value
Age (years), mean (SD)	75.96 (15.82)	75.47 (16.31)	76.46 (15.32)	0.855
< 75, n (%)	186 (35.8)	95 (36.3)	91 (35.4)	
Sex (male: female), n (%)	321:198 (61.8:38.2)	160:102 (61.1:38.9)	161:96 (62.6:37.4)	0.719
Location of cardiac arrest				0.148
Home, n (%)	377 (72.6)	185 (70.6)	192 (74.7)	
Inside public property, n (%)	63 (12.1)	40 (15.3)	23 (8.9)	
Outside public property, n (%)	62 (11.9)	28 (10.7)	34 (13.2)	
In-ambulance, n (%)	17 (3.3)	9 (3.4)	8 (3.1)	
Witness, n (%)	193 (37.2)	99 (37.8)	94 (36.6)	0.786
Bystander CPR, n (%)	130 (25.0)	80 (30.5)	50 (19.5)	0.004
Bystander CPR by location				
Home, n (%)		45 (24.3)	26 (13.5)	0.008
Inside public property, n (%)		15 (37.5)	9 (39.1)	1
Outside public property, n (%)		28 (10.7)	34 (13.2)	0.191
In-ambulance, n (%)		8 (88.9)	5 (71.4)	0.55
AED use, n (%)	24 (4.6%)	14 (5.3)	10 (3.9)	0.532
Shockable rhythm, n (%)	40 (7.7%)	25 (9.5)	15 (5.8)	0.139
EMS response time (min.)				
EMS call to scene (min.), mean (SD)	7.79 (4.66)	7.21 (5.55)	8.38 (3.43)	0.004
Scene to contact (min.), mean (SD)	1.99 (3.42)	1.77 (1.61)	2.21 (4.57)	0.143
Contact to departure (min.), mean (SD)	11.86 (4.83)	11.56 (4.46)	12.17 (5.18)	0.151
Departure to arrival (min.), mean (SD)	9.06 (3.69)	8.64 (3.18)	9.49 (4.10)	0.009
EMS call to arrival (min.), mean (SD)	30.81 (9.02)	29.40 (8.91)	32.25 (8.92)	< 0.001
Adrenaline administration, n (%)	351 (67.6)	168 (64.1)	183 (71.2)	0.092
Time of first adrenaline administration (min.), mean (SD)	35.60 (9.51)	33.30 (8.23)	37.70 (10.13)	< 0.001
COVID-19 infection, n (%)	2 (0.4)	0 (0)	2 (0.8)	0.059
ROSC, n (%)	122 (23.5)	73 (27.9)	49 (19.1)	0.023
Admission, n (%)	52 (10.0)	33 (12.6)	19 (7.4)	0.057
Discharge, n (%)	16 (3.1)	8 (3.1)	8 (3.1)	1
CPC 1–2 at discharge, n (%)	8 (0.02)	5(0.01)	6 (0.01)	1
CPC 3–4 at discharge, n (%)	8 (0.02)	3(0.01)	2 (0.01)	1

*OHCA* out-of-hospital cardiac arrest; COVID-19, coronavirus disease 2019, *SD* standard deviation, *CPR* cardiopulmonary resuscitation, *AED* automated external defibrillator, *EMS* emergency medical services, *ROSC* return of spontaneous circulation, *CPC* cerebral performance category.

Table 2 shows the statistically significant differences in the univariate analysis of the various outcomes. ROSC was statistically significantly associated with shockable rhythm (OR 6.27, 95% CI 1.52–25.9), presence of witnesses (OR 5.18, 95% CI 1.74–15.4), and time from calling EMS to arrival (OR 0.85, 95% CI 0.76–0.97). Unlike the results of the multivariate analysis described below, ROSC was not statistically significantly associated with the intra-pandemic period in the univariate analysis. Survival to admission was associated with the provision of bystander CPR (OR 7.78, 95% CI 2.29–26.4), shockable rhythm (OR 10.0, 95% CI 2.51–39.8), and presence of witnesses (OR 9.67, 95% CI 2.41–38.7). Survival to discharge was associated with shockable rhythm (OR 7.05, 95% CI 1.04–47.8), and good neurological prognosis (CPC1–2) was associated with adrenaline administration (OR 18, 95% CI 0.24–261).

**Table 2:** Univariate analysis for the odds ratios of outcomes in the pre-pandemic and intra-pandemic periods.

Outcome	Factor	Odds ratio (95% CI)	*P* value
ROSC	Shockable rhythm	6.27 (1.52–25.9)	0.0011
	Witness	5.18 (1.74–15.4)	0.0003
	EMS call to arrival (min)	0.85 (0.76–0.97)	0.0012
Admission	Bystander CPR	7.78 (2.29–26.4)	0.0001
	Shockable rhythm	10.0 (2.51–39.8)	0.0001
	Witness	9.67 (2.41–38.7)	0.0001
Survival	Shockable rhythm	7.05 (1.04–47.8)	0.0046
CPC 1–2	Adrenaline administration	18 (1.24 –261)	0.0034

*CI* confidence interval, *ROSC* return of spontaneous circulation, *EMS* emergency medical services, *CPR* cardiopulmonary resuscitation, *CPC* cerebral performance category.

The following independent and dependent variables were used for logistic regression analysis; provision of AED, provision of prehospital electrical defibrillation, age, provision of bystander CPR, presence of COVID infection, cause of death, place of cardiac arrest, occurring during the intra-pandemic, sex, whether the initial waveform was a shockable rhythm or not, presence of witnesses, EMS response time, adrenaline administration, and time of first adrenaline administration. Dependent variables were ROSC, survival at admission, survival at discharge, and good neurological prognosis (CPC 1–2) at discharge.

Only items with *p* values < 0.05  are listed.

Table 3 shows the results of the multivariate analysis of the outcomes, with a cut-off time of 30 min for the first adrenaline administration. ROSC was significantly associated with the intra-pandemic period (OR 0.56, 95% CI 0.33–0.94), shockable rhythm (OR 6.26, 95% CI 2.53–15.50), and presence of witnesses (OR 2.58, 95% CI 1.54–4.33). Survival to admission was associated with age (> 75 years; OR 0.09, 95% CI 0.02–0.27), shockable rhythm (OR 16.4, 95% CI 5.32–50.90), presence of witnesses (OR 2.84, 95% CI 1.13–7.11), and time from calling EMS to arrival (OR 0.92, 95% CI 0.86–0.98). Survival to discharge was statistically significantly associated with shockable rhythm (OR 11.3, 95% CI 1.99–64.5) and first adrenaline administration within 30 min (OR 15.6, 95% CI 1.74–140). We performed analyses with cut-off values of 10, 20, and 35 min for the time of first adrenaline administration, and similar results were observed. The median duration of adrenaline administration in our patient group was 35 min. The time of the first adrenaline administration was not associated with any of the outcomes (Supplementary Table S1).

**Table 3:** Logistic regression analysis for the odds ratios of outcomes (ROSC, admission, and survival) before and during the pandemic.

Outcome	Factor	Odds ratio (95% CI)	*P* value
ROSC	Pandemic	0.56 (0.34–0.94)	0.0029
	Shockable rhythm	6.26 (2.53–15.5)	< 0.0001
	Witness	2.58 (1.54–4.33)	0.0003
Admission	Age > 75 years	0.09 (0.03–0.28)	< 0.0001
	Shockable rhythm	16.4 (5.32–50.9)	< 0.0001
	Witness	2.84 (1.13–7.11)	0.0026
	EMS call to arrival (min)	0.92 (0.86–0.98)	0.0015
Survival	Shockable rhythm	11.3 (1.99–64.5)	0.0006
	First adrenaline administration time < 30 min	15.6 (1.74–140)	0.0014

*ROSC* return of spontaneous circulation, *CI* confidence interval, *EMS* emergency medical services.

The following independent and dependent variables were used for logistic regression analysis; provision of AED, provision of prehospital electrical defibrillation, age, provision of bystander CPR, presence of COVID infection, cause of death, place of cardiac arrest, occurring during the intra-pandemic, sex, whether the initial waveform was a shockable rhythm or not, presence of witnesses, EMS response time, adrenaline administration, and time of first adrenaline administration. Dependent variables were ROSC, survival at admission, survival at discharge, and good neurological prognosis (CPC 1–2) at discharge.

Only items with *p* values < 0.05 are listed.

Table 4 shows the results of the subgroup analysis for the shockable rhythm and non-shockable rhythm groups. The logistic regression analysis for each outcome showed statistically significant differences in variables such as age (> 75 years) and sex, while there were no statistically significant associations in survival to discharge in the shockable rhythm group.

**Table 4:** Logistic regression analysis for the odds of outcomes in the shockable rhythm and non-shockable rhythm groups.

Outcome	Factor	Odds ratio (95% CI)	*P* value
Non-shockable rhythm group (n = 479)			
ROSC	Pandemic	0.48 (0.28–0.83)	0.0009
	Witness	2.97 (1.72–5.13)	< 0.0001
Admission	AED	15 (2.37–95.7)	0.0004
	Age > 75 years	0.08 (0.02–0.34)	0.0001
	EMS call to arrival (min)	0.91 (0.84–0.98)	0.0015
Shockable rhythm group(n = 40)			
ROSC	Female	0.13 (0.02–0.94)	0.0044
Admission	Age > 75 years	0.14 (0.02–0.79)	0.0026

*CI* confidence interval, *ROSC* return of spontaneous circulation, *AED* automatic external defibrillator, *EMS* emergency medical services.

The following independent and dependent variables were used for logistic regression analysis; provision of AED, provision of prehospital electrical defibrillation, age, provision of bystander CPR, presence of COVID infection, cause of death, place of cardiac arrest, occurring during the intra-pandemic, sex, presence of witnesses, EMS response time, adrenaline administration, and time of first adrenaline administration. Dependent variables were ROSC, survival at admission, survival at discharge, and good neurological prognosis (CPC 1–2) at dischare.

Only items with *p* values < 0.05are listed.

Table 5 shows the results of the subgroup analysis comparing the presence and absence of witnesses. In the witness group, ROSC was associated with the intra-pandemic period (OR 0.42, 95% CI 0.21–0.87) and shockable rhythm (OR 3.09, 95% CI 1.03–9.23). Survival to admission was significantly associated with age (> 75 years; OR 0.14, 95% CI 0.02–0.53), the intra-pandemic period (OR 0.18, 95% CI 0.05–0.70), and shockable rhythm (OR 14.4, 95% CI 3.91–52.7).

**Table 5:** Logistic regression analysis for the odds of outcomes in the witness and non-witness groups.

Outcome	Factor	Odds ratio (95% CI)	*P* value
Non-witness group (n = 326)			
ROSC	Shockable rhythm	17.3 (3.33–89.8)	0.0001
Admission	Age > 75 years	0.02 (0.01–0.28)	0.0004
	Female	6.23 (1.27–30.5)	0.0024
	Shockable rhythm	95.1 (7.41–1220)	< 0.0001
Witness group (n = 193)			
ROSC	Pandemic	0.42 (0.21–0.87)	0.0020
	Shockable rhythm	3.09 (1.03–9.23)	0.0021
Admission	Age > 75 years	0.14 (0.02–0.53)	0.0002
	Pandemic	0.18 (0.05–0.70)	0.0024
	Shockable rhythm	14.4 (3.91–52.7)	< 0.0001
Survival	Shockable rhythm	15.1 (1.3–176)	0.0030

*CI* confidence interval, *ROSC* return of spontaneous circulation.

The following independent and dependent variables were used for logistic regression analysis; provision of AED, provision of prehospital electrical defibrillation, age, provision of bystander CPR, presence of COVID infection, cause of death, place of cardiac arrest, occurring during the intra-pandemic, sex, presence of witnesses, EMS response time, adrenaline administration, and time of first adrenaline administration. Dependent variables were ROSC, survival at admission, survival at discharge, and good neurological prognosis (CPC 1–2) at dischare.

Only items with *p* values < 0.05 are listed.

## Discussion

There was a statistically significant decrease in bystander CPR, prolongation of transport time, and delay in first adrenaline administration during the intra-pandemic, compared to that in the pre-pandemic period. The results of this study suggest that the COVID-19 pandemic reduced the number of ROSCs of patients with OHCA through prolonged transport time and delayed first adrenaline administration time. Although not shown in this study, it may have reduced survival through the reduction of ROSCs, and further large-scale studies are needed.

The COVID-19 pandemic may have included a wide variety of factors that affect the prognosis of OHCA. Previous studies have also suggested that the COVID-19 pandemic possibly worsened the prognosis of OHCA patients through causes other than known confounders^12^, such as changes in social life, medical history, and COVID-19-related medical practice. Although we could not examine them in this study, these are areas of consideration for future research.

In the EMS protocol in Yokohama city, chest compression was started after covering the mouth and nose of the patient with a surgical mask. At Yokohama City Hospital, where our study was conducted, chest compressions were stopped during intubation to prevent infection due to aerosol production. These may have affected the resuscitation rate.

The results of the two-group comparison showed a decrease in bystander CPR in the prehospital item comparison, but contrary to expectations, there was no change in the breakdown of where the cardiopulmonary arrests occurred or in the percentage of AED use. This result contradicts previous studies reporting a decrease in OHCA in public places and an increase in private areas^2,4^.

In this study, there was a statistically significant decrease in bystander CPR. When bystander CPR rates were compared by location, only OHCA occurring at home was significantly lower during the intra-pandemic period. Since the number of cardiac arrests in the home itself has increased, the decrease in bystander CPR rates at home may have contributed to the overall decrease in bystander CPR rates. In previous studies, bystander behavioural changes were cited as the reason for the decrease in bystander CPR during the COVID-19 pandemic. Patient contact and aerosol production from chest compressions are pathways for COVID-19 infection^13^, and bystanders who do not have the expertise may also choose not to perform CPR due to the fear of infection. However, this may not necessarily be true for those who only experienced bystander CPR in their homes in our study. In Japan, where the population is aging and the birth rate is declining, older adults are likely to be home alone, in which case bystander CPR cannot be performed. Restrictions on going out during the COVID-19 pandemic may have spurred this trend by causing isolation among elderly households.

The time between calls to EMS and arrival at the hospital was prolonged during the intra-pandemic period. Shockable rhythms were less frequently observed during COVID-19. The results of this study suggest that the COVID-19 pandemic reduced shockable rhythms through prolonged transport time. The time was divided into four sections: call to the arrival of EMS at the scene, arrival of EMS at the scene to patient contact, patient contact to departure, departure, and arrival at the hospital; the time was prolonged in two sections: EMS call to scene and arrival at the hospital. The time between emergency calls and the arrival of EMS at the scene was extended by 1 min on average, and similar results were found in studies from Paris and Taiwan^14,15^. The number of emergency calls in Yokohama City was 194,639 during the pre-pandemic period and 159,049 during the intra-pandemic period, with a decrease observed in the intra-pandemic period^16^. This contradicts the findings of previous studies that showed an increase in emergency calls during the COVID-19 pandemic^17,18^. One of the reasons for the prolonged response time despite the decrease in the number of calls is the time required to put on PPE. Previous studies have also suggested that wearing PPE leads to longer transport times^19^. PPE is necessary to prevent COVID-19 infection^20^, and the equipment used by emergency teams during OHCA responses has been changed in Yokohama City. Specifically, before the pandemic, only a jumper protective suit was worn on the upper half of the body, but during the pandemic, PPE comprising a jacket and pants was additionally worn. Regarding the delayed hospital arrival time, it is possible that the hospital of choice became more distant during the pandemic period or that traffic congestion due to the increased use of private vehicles in consideration of the risk of public transport-associated infection may have had an effect.

A previous study comparing the risk of infection and the lifesaving rate of CPR in COVID-19-infected patients stated that delaying bystander CPR to wear PPE should only be considered if there is a significant increase in the prevalence of COVID-19^21^. However, it would be difficult to establish specific criteria for changing the protocol when the prevalence increases by a specific amount, and when in reality, PPE should be worn in all cases.

In this study, time to adrenaline administration was prolonged during the intra-pandemic period, and adrenaline administration within 30 min was significantly associated with survival to discharge. The latest guidelines recommend the administration of adrenaline as early as possible^22,23^. A recent randomized controlled trial and two meta-analyses showed that the use of epinephrine improved the rate of survival to hospital discharge in OHCA patients^24–26^. Although there is no consensus on the timing of adrenaline administration, there are reports that adrenaline administration in less than 15 min is significantly associated with ROSC^27^. The early administration of epinephrine (≤ 10 min) was also associated with a favourable neurological outcome in adult bystander-witnessed OHCA^28,29^. However, there are studies that report poor survival outcomes after administration of epinephrine during CPR^30^. There are controversies regarding the survival benefits of administration of epinephrine during CPR.

### Limitations

Some limitations to this study should be noted. First, we may not have adequately controlled for confounding factors, such as staffing, which may have influenced patient outcomes in relation to the COVID-19 pandemic. Second, we have not been able to measure the duration of CPR interruptions due to changes in protocols, which may have worsened the quality of CPR. Additionally, from our retrospective data analysis we cannot tell about the frequency of intermediate ROSC and whether CPR was performed during transport.

Third, although a sufficiently low *p* value was detected, our study also used a statistical treatment with a significance level of 0.05 for multiple testing. In addition, our study does not reflect data on deaths of patients that were not transported to a medical institution, because death was obvious at the time of discovery.

Fourthly, the number of deaths in Yokohama City increased by 1605, from 33,019 during the pre-pandemic period to 34,624 during the intra-pandemic period. This number may include COVID-19 influenced deaths; for example, the isolation of elderly people living alone or changes in the pathology of background diseases due to less frequent hospital visits. In addition, the data used were from a single institution (Yokohama Municipal Hospital); hence, the findings of this study may not be generalizable to other regions or institutions. Fifthly, since this was an observational study, we could not suggest any causal relationship. Further research is required to enhance prehospital care.

## Conclusions

The COVID-19 pandemic possibly worsened the short- and long-term prognoses of patients with OHCA through various factors such as decreased bystander CPR, prolonged transport time, and time to first adrenaline administration. Significant factors affecting the prognosis of OHCA patients could be mitigated by medical personnel who could shorten the transport time and time to adrenaline administration by securing the intravenous route early. However, this was a single-centre study, and multicentre studies are required to validate these findings.

## Methods

### Study design

We conducted a retrospective cohort study of adult OHCA patients aged ≥ 18 years who were transported to Yokohama Municipal Citizen’s Hospital by EMS between January and December 2019 before COVID-19 pandemic (pre-pandemic group) and between April 2020 and March 2021 during the pandemic (intra-pandemic group), forming the pre-pandemic and intra-pandemic groups, respectively. Cases reported from January to March 2020 were excluded due to being in the transition period. Patients with ROSC at the time of EMS contact, patients with confirmed Do Not Attempt Resuscitation (DNAR) by EMS orders, patients transferred from other medical facilities, and patients with exogenous OHCA—defined as those whose primary cause of death was one of the following: trauma, drowning, hanging, suffocation, or burns—were excluded from the study. We obtained approval from the Ethical Review Committee (Yokohama municipal citizen’s hospital) and permission from the head of the research institution before conducting this study. This study is a retrospective study using existing data. To protect patient's privacy, each patient is assigned a research subject code so that patients cannot be identified from the research subject code. This research did not involve the acquisition of new samples or information and was conducted using only existing information. Since it was difficult to obtain written informed consent from the research subjects, information about the research was disclosed to them via the website of Yokohama Municipal Hospital, and the research subjects were provided the opportunity to refuse participation in the research being conducted. The study was conducted in accordance with the principles of the Declaration of Helsinki, per the Personal Information Protection Law and National Research Ethics Guideline in Japan.

The derivation group data were collected by one emergency physician from electronic medical charts. The validation cohort data were prospectively collected by resident physicians after they received a short lecture on OHCA.

### Study setting and population

Yokohama Municipal Hospital is located in Yokohama City, which has a population of approximately 3.78 million people, and the cumulative number of COVID-19 infected persons was 99,955 as of 31 January 2022^27^. During the intra-pandemic (April 2020–March 2021), the country experienced three waves of infections, in April–May 2020, August 2020, and January–February 2021, with the third wave being the largest. The highest number of new infections was 985 per day (15 January 2021), and the highest number of new cases per week was 66.24 per 100,000 people (12–18 January 2021)^31,32^. A state of emergency was declared for a total of 132 days from 7 April 2020 to 25 May 2020, and from 8 January 2021 to 21 March 2021 (the lockdown policy mainly restricted the use of restaurants).

EMS in Yokohama city basically treat the OHCA patients according to the Japanese resuscitation guidelines published from the Japan Resuscitation Council (JRC)^33^ which are developed based on the statements from the International Liaison Committee on Resuscitation (ILCOR)^34^. That includes provision of Intravenous access, adrenaline administration, airway management using laryngeal tube, and manual chest compressions. No mechanical CPR devices were deployed by EMS during the study period. During the COVID-19 infection-spreading period, the EMS in Yokohama city implemented a protocol to treat all the cardiac arrest patients as possibly having COVID-19. In the EMS protocol, before entering the scene, all staff donned PPE that included N95 masks and eye protection, and a high-efficiency particulate air (HEPA) filter was attached securely to any manual or mechanical ventilation device in the path of exhaled gas. Chest compression was started after covering the mouth and nose of the patient with a surgical mask.

At Yokohama City Hospital, where our study was conducted, chest compressions were stopped during intubation to prevent infection due to aerosol production.

### Study protocol and measures

The following prehospital and post-surgical information was collected from the transport and medical charts: age (75 years or older or younger), sex, place of occurrence (home, indoor public space, outdoor public space, or ambulance), presence or absence of witnesses, presence or absence of bystander CPR, rate of bystander CPR by place of occurrence, AED use, initial waveform (shockable or non-shockable), EMS response time {EMS call to scene time (from call to arrival on scene), Scene to contact time (arrival on scene to contact), Contact to departure time(on scene emergency care by EMS), Departure to arrival time (from scene to hospital), EMS call to arrival time (from call to arrival hospital)}, prehospital adrenaline administration, initial adrenaline administration time, cause of death (cardiogenic, noncardiogenic, extrinsic), presence of ROSC, survival at hospitalization, survival at discharge, and good neurological prognosis (CPC1–2) at discharge.

The EMS team was required to wear PPE during the pandemic period to prevent airborne infection. Emergency teams can provide advanced airway clearance, venous route clearance, and adrenaline administration by tracheal intubation or use of a laryngeal tube for patients with cardiac arrest.

### Data analysis

All variables were compared during the intra-pandemic and pre-pandemic periods. Categorical variables are presented as frequencies (%) and were compared using Fisher’s exact test. Continuous variables are presented as means (standard deviation) and were compared using the Mann–Whitney U test. In addition, the odds ratio (OR) and 95% confidence interval (CI) were calculated to determine the effect of the pandemic after adjusting for relevant factors using a multivariate logistic regression model with a variable reduction method, and factors other than known confounders were examined. Two subgroup analyses were performed with patient groups divided according to the initial waveform and the presence of witnesses.

Four cut-off values of 10, 20, 30, and 35 min for time to adrenaline administration were used for the sensitivity analysis. Patients were considered cardiogenic if a non-cardiogenic disease (cerebrovascular disease, respiratory disease, or malignancy) could be excluded from the clinical course and laboratory findings.

The following independent and dependent variables were used for logistic regression analysis. Independent variables included provision of AED, provision of prehospital electrical defibrillation, age, provision of bystander CPR, presence of COVID infection, cause of death, place of cardiac arrest, occurring during the intra-pandemic, sex, whether the initial waveform was a shockable rhythm or not, presence of witnesses, EMS response time, adrenaline administration, and time of first adrenaline administration. Dependent variables were ROSC, survival at admission, survival at discharge, and good neurological prognosis (CPC 1–2) at discharge.

All tests were two-tailed and considered statistically significant at *p* < 0.05. All statistical analyses were performed using EZR (Saitama Medical Center, Jichi Medical University, Saitama, Japan), a graphical user interface for R (The R Foundation for Statistical Computing, Vienna, Austria)^35^. More precisely, it is a modified version of R commander designed to add statistical functions frequently used in biostatistics.

### Supplementary Information

Supplementary Information.

## Data Availability

The datasets generated and/or analysed during the current study are not publicly available due to the restriction by IRB, but are available from the corresponding author on reasonable request.
