# Supplementary material for: Out-of-hospital cardiac arrest patients during the coronavirus disease 2019 pandemic
Source: Sci Rep. 2023 Dec 27;13:23005. doi: 10.1038/s41598-023-50150-z (PMC10754886; doi:10.1038/s41598-023-50150-z)
Supplement: Supplementary file 1 — Supplementary Information. [file 41598_2023_50150_MOESM1_ESM.docx]

**Supplementary Table S1. Logistic regression analysis for the odds of outcomes before and during the COVID-19 pandemic, using cut-off values of 10, 20, and 35 min for the time of first adrenaline administration**

| Outcome | Factor | Odds ratio (95% CI) | P-value |
| --- | --- | --- | --- |
| ROSC | Pandemic | 0.56 (0.34–0.94) | 0.0029 |
|  | Shockable rhythm | 6.26 (2.53–15.5) | <0.0001 |
|  | Witness | 2.58 (1.54–4.33) | 0.0003 |
| Admission | Age >75 years | 0.09 (0.03–0.28) | <0.0001 |
|  | Shockable rhythm | 16.4 (5.32–50.9) | <0.0001 |
|  | Witness | 2.84 (1.13–7.11) | 0.0026 |
|  | EMS call to arrival (min.) | 0.92 (0.86–0.98) | 0.0015 |
| Survival | Shockable rhythm | 11.3 (1.99–64.5) | 0.0005 |
|  | EMS call to arrival (min) | 0.79 (0.67–0.96) | 0.0017 |

＊The results of each analysis were similar, as shown in the table.

Abbreviations: COVID-19, coronavirus disease 2019; ROSC, return of spontaneous circulation; CI, confidence interval; EMS, emergency medical service

K.W., K.M., K.S., T.A., S.I., and I.T. were involved in study design, data collection and data interpretation. K.W., K.M.,and T.A. were involved in the data analysis. All authors critically revised the report, commented on drafts of the manuscript, and approved the final report.

• The datasets generated and/or analysed during the current study are not publicly available due to the restriction by IRB, but are available from the corresponding author on reasonable request.
